# Controversy around climate change reports: a case study of Twitter responses to the 2019 IPCC report on land

**DOI:** 10.1007/s10584-021-03182-1

**Published:** 2021-08-31

**Authors:** Mary Sanford, James Painter, Taha Yasseri, Jamie Lorimer

**Affiliations:** 1 Oxford Internet Institute, University of Oxford, 1 St Giles, Oxford, OX1 3JS UK; 2 Reuters Institute for the Study of Journalism, University of Oxford, 13 Norham Gardens, Oxford, OX2 6PS UK; 3 School of Geography and Environment, University of Oxford, S Parks Rd, Oxford, OX1 3QY UK; 4 School of Sociology, University College Dublin, Belfield, Dublin 4, Ireland; 5 Geary Institute for Public Policy, University College Dublin, Belfield, Dublin 4, Ireland

**Keywords:** Climate change, IPCC, Twitter, Diet, Contention, Content analysis

## Abstract

In August 2019, the Intergovernmental Panel on Climate Change (IPCC) published its Special Report on Climate Change and Land (SRCCL), which generated extensive societal debate and interest in mainstream and social media. Using computational and conceptual text analysis, we examined more than 6,000 English-language posts on Twitter to establish the relative presence of different topics. Then, we assessed their levels of toxicity and sentiment polarity as an indication of contention and controversy. We find first that meat consumption and dietary options became one of the most discussed issues on Twitter in response to the IPCC report, even though it was a relatively minor element of the report; second, this new issue of controversy (meat and diet) had similar, high levels of toxicity to strongly contentious issues in previous IPCC reports (skepticism about climate science and the credibility of the IPCC). We suggest that this is in part a reflection of increasingly polarized narratives about meat and diet found in other areas of public discussion and of a movement away from criticism of climate science towards criticism of climate solutions. Finally, we discuss the possible implications of these findings for the work of the IPCC in anticipating responses to its reports and responding to them effectively.

**Supplementary Information:**

The online version contains supplementary material available at 10.1007/s10584-021-03182-1.

## Introduction

For millions of people around the world, social media are increasingly important entrances to information and act as major mediators of public opinion formation. Results from online surveys in 37 countries in 2018 found that for nearly a quarter (23%) of those surveyed, social media were the main way they came across news (Newman et al. 2018). Of these, the microblogging service Twitter has attracted millions of users since its creation in 2006. Along with several other social media platforms, it has become a major and controversial tool for social and political action (Cihon and Yasseri 2016). Its openness and accessibility has led it to become one of the top social media for people around the world to connect, exchange ideas, raise awareness, but also to debate, slander, and sometimes radicalize other users (Kwak et al. 2010; Yasseri and Menczer 2021). It has given life to several major social and political movements including the Arab Spring of 2011 and the ongoing MeToo movement. It also plays a central role in politics and policy discourse (Shapiro and Hemphill 2017).

In the climate change domain, Twitter has played a major role in facilitating dialogue and debate between activists, skeptics, and more neutral users (Williams et al. 2015; Holmberg and Hellsten 2016; Garcia et al. 2019). In particular, reports from the Intergovernmental Panel on Climate Change (IPCC) are known to prompt a high volume of social media responses (Kirilenko and Stepchenkova 2014; O’Neill et al. 2015) and of sentiment polarization (Pearce et al. 2014). Both on mainstream and social media, this polarized debate has tended to focus mostly on the reality of climate change, the validity or robustness of climate science, the solutions (including the need to take any action), and the credibility of the IPCC (Jang and Hart 2015; O’Neill et al. 2015).

Recent studies point to high levels of incivility and sentiment polarity on social media around a variety of issues including the SARS COVID-19 pandemic (Majó-Vázquez et al. 2020), political personalities in the 2016 US elections (Zheng and Shahin 2018), the Brexit debate in the UK (Del Vicario et al. 2017), and immigration in Germany (Humprecht et al. 2020). User comments on social media about climate change also frequently contain high levels of negative emotions, rudeness and incivility, and anti-political rhetoric (Pearce et al. 2014; Olausson 2019). For example, Twitter discussions on climate change show patterns of sarcasm and incivility amongst those holding skeptical perspectives on climate change and right-leaning politics (Anderson and Huntingdon 2017). More generally, social media analysis can also offer important insights into wider popular opinion and debate about environmental issues (Tandoc and Eng 2017; Olausson 2018) and climate change in particular (Pearce et al. 2018). This includes the relative presence of different topics found in tweets (Newman 2017).

We chose for our analysis the response on Twitter to the IPCC Special Report on Climate Change and Land (SRCCL) launched on 8 August 2019 (IPCC 2019). There are several reasons for this. First, it included extensive discussion of the options for reducing greenhouse gas (GHG) emissions from agricultural systems, including reducing meat consumption and other dietary options. Recent research has stressed the value of lessening individual meat consumption for achieving reductions in GHG emissions from the sector (Poore and Nemecek 2018) and the need for a transformation in eating habits to improve health within environmental targets (Willett et al. 2019). The nexus of climate change, land, and diet is an increasingly important issue in the media and public sphere and subject to competing narratives and counter-narratives (Kristiansen et al. 2020; Maye et al. 2021), with considerable push back evident from those opposed either to reductions in meat eating or to no meat eating at all (e.g. Garcia et al. 2019).

Secondly, on Twitter alone, the report generated significant discussion. Using the Twitter Application Programming Interface (API), we collected an initial sample of over 27,000 tweets in 41 languages during the month of August, which gave us a robust dataset to analyze. In addition to the dietary question, news media also reported on a variety of issues found in the report (Sauer 2019), which gave organizations and individuals different options to discuss. Finally, the release of the SRCCL contributed to a historically high volume of international coverage of climate change in the media in August 2019 (Boykoff et al. 2020).

Within this context, we were particularly interested in determining which of the topics found in the SRCCL Twitter users responded to or talked about, and the extent to which these topics differed from the official priority messages of the SRCCL and from previously prevalent frames and public disputes around IPCC reports. The Twitter data were analyzed to explore the levels of contention about different aspects of the report, as measured by toxicity and sentiment polarity, with a particular focus on the discussion about dietary change and meat eating. In this way, we could assess the extent to which competing narratives prevalent on social media around diet, meat-eating, and climate change dominated the response to the SRCCL, forging a debate and level of dispute markedly different to the main content and communication priorities of the IPCC report. Our analysis highlights the importance and shortcomings of social media-based public debates in response to major public policy events.

## Background literature

### Meat in the public sphere

In recent years, there has been a discernible shift in the historical position of veganism at the margins of many cultures, which some scholars have described as the “mainstreaming” of veganism (Sexton et al. forthcoming; Giraud 2021). There has been an unprecedented growth in sales of vegan products in the USA and Europe (Parker 2018); the number of declared vegans has increased significantly; new vegan products are regularly announced by manufacturers and supermarkets; vegan hashtags have become highly used on Instagram, and vegan celebrities and influencers have become prominent on social media (Sexton et al. forthcoming).

This growing interest in veganism (and eating less meat or “flexitarianism”) has coincided with a series of widely publicized reports associating meat eating and livestock farming with a range of negative impacts, particularly on GHG (methane) emissions (McGregor et al. 2021) and other environmental concerns such as biodiversity, land use, and planetary boundaries (Godfray et al. 2018; Springmann et al. 2018; Poore and Nemecek 2018). The 2019 Eat-Lancet Report placed a particular emphasis on reducing the consumption of red meat both for health and environmental reasons (Willett et al. 2019).

Mainstream and social media have begun to highlight the negative impacts of meat eating (Maye et al. 2021). For example, research into mainstream media coverage in the USA and UK showed that historically the amount of attention paid to the climate change-animal agriculture link was modest compared to the focus on other drivers of emissions such as transport and energy, but this began to change in 2018 with the publication of the reports like those mentioned above and in 2019 with the launch of the IPCC’s SRCCL (Kristiansen et al. 2020).

Research also shows that while the vegan mainstreaming and the rise of plant-based meat and dairy alternatives have often been framed in the media, corporate and advocacy sectors through a positive narrative with multiple benefits for animals, health, and the environment (Clay et al. 2020), other industry-based or NGO sectors, such as the National Farmers Union and the Sustainable Food Trust in the UK, have pushed back against some of these narratives with counter-narratives of their own (Sexton et al. 2019; Maye et al. 2021).

Although analysis of these debates about meat eating on social media has historically been limited (Kristiansen et al. 2020), this has begun to change. Goodman and Jaworska (2020) showed that a growing band of “Digital Food Influencers” (who construct, curate, and share the meanings of good food) on Twitter create prominent “good food grammars” related to clean eating and lifestyle, which includes eating no meat (p. 189).

The issue has become subject to a strong polarization of views on social media. For example, analysis of Facebook users in Sweden showed that the discussion of meat eating was legitimized in part through a polarization between livestock production and other (environmental) issues such as vacation travel (Olausson 2018). Maye et al. (2021) situated their research into Twitter narratives around meat within the concept of “online issue publics” who have emerged as part of different story-networks celebrating or disputing meat eating (Lee et al. 2014). However, of central relevance to our research, these polarized debates about meat eating on social media can be prompted by landmark, science-based reports given particular prominence in mainstream media. In the case of the Eat-Lancet Report, published before the SRCCL in January 2019, research on more than eight million Twitter posts commenting on it showed that the report “prompted highly polarized debates online including misinformation, conspiracy theories, and personal attacks along with the hashtag #yes2meat” (Garcia et al. 2019, p. 2153). In the months following the report, critics of the planetary health diet reached 26 million people on Twitter and came to dominate the online discussion. Such findings suggest a fruitful line of inquiry into whether similar levels of contention can be seen in response to the IPCC report, even though the focus, authorship, and purpose of the reports were different.

### IPCC reports and controversy in the media

IPCC reports play a central role in summarizing and presenting climate science, creating media coverage, and affecting public awareness. They exert a wide-ranging influence on how climate science interacts with policy (O’Neill et al. 2015; Bjurström and Polk 2011) and in particular provide the scientific basis for the decisions coming out of the main international forum for policy formation on climate change, the UNFCCC.^1^ The summaries for policy makers (SPMs) for each IPCC report have been seen as transferring knowledge from experts in one field to policy makers and experts in other fields (Barkemeyer et al. 2016). They communicate headline scientific messages to publics around the world, particularly via the news media (Kunelius et al. 2017).

Previous studies show how mainstream and social media play a major role in shaping or framing the way IPCC reports are presented and received by audiences (Hulme 2009; Painter 2013; Painter 2014; Pearce et al. 2014; O’Neill et al. 2015). In particular, research has highlighted how IPCC reports have been subject to various forms of contention. For example, O’Neill et al. (2015) found that the Working Group 1 report (WG1) of the IPCC’s Fifth Assessment Report (AR5) was often contested and politicized in television and print reporting in the USA and UK. This often centered on the presence or absence of the “settled science” or “uncertain science” frames, the latter being defined as “a focus on uncertainty—in climate science, impacts, or solutions. Many question the anthropogenic nature of climate change or discuss natural variability” (O’Neill et al. 2015, p. 381). Although there were important differences between countries and media outlets as to the prevalence of the “uncertain science” frame, Twitter response to the AR5 was dominated by the “settled science” frame.

In similar fashion, Painter (2014) found that the uncertainty frame was present in nearly 90% of the print media reporting in six countries of the WG1 and WG2 of the IPCC’s 2007 AR4 report and of the IPCC’s 2012 report on extreme weather events. In this case, the uncertainty frame included uncertainty about climate science (for example, about the timing and extent of climate impacts), but also the presence of climate skeptics.

The presence of skeptics on social media has also been widely analyzed and is a major driver of contention there. For example, Jang and Hart (2015) showed that certain frames which promoted climate skepticism were widely circulated by Twitter users, within specific regional and political contexts. Examining Twitter messages geographically based in four English-speaking countries (the USA, UK, Canada, and Australia) from mid-2012 to mid-2014, the authors found that “hoax frames” which question the reality of climate change were particularly common in predominately Republican “red” states in the USA, compared to the UK, Canada, and Australia, and predominantly Democrat “blue” states. Hoax elements were also included in a Politics/Hoax topic that was identified as one of the most common in nearly 400,000 tweets from users in the USA, UK, Canada, and Australia from mid-2016 to early 2018 (Dahal et al. 2019).

Pearce et al. (2014) showed that Twitter responders to the WG1 of AR4 varied according to geographical location and the degree to which they were supportive, unsupportive, or neutral in their tweets about the IPCC. More than half of the 239 Twitter users they analyzed were coded as “supportive” (i.e., broadly either of the climate science or of measures to reduce carbon emissions), roughly a quarter was “unsupportive” of, or unconvinced by, climate science or policies, and the rest were neutrals (i.e., not taking a stance). Newman (2017) included analysis of the relative presence of different “issue focuses” found in the 100 most retweeted posts in response to the IPCC’s WG1 of AR5. The author found that uncertainty, which in this case meant any tweet that discussed the dismissal or questioning of the report’s conclusions, represented about 6% of the posts.

Another form of skepticism historically found in mainstream and social media has stressed what it sees as the general lack of credibility of the IPCC. Skeptics have argued that it is dominated by like-minded scientists accepting mainstream climate science and rejecting outliers, or they have targeted prominent IPCC authors for supposed scientific malpractice.

The widespread presence of skeptical individuals and groups on social media has prompted research into the degree to which Twitter exacerbates already polarized public opinion. Williams et al. (2015) examined tweet responses to the IPCC’s AR5 report and found that social networks were characterized by strong attitude-based homophily and segregation into polarized “activist” groups (holding views consistent with the scientific consensus and/or promoting action to prevent climate change) and “sceptic” groups (holding views in opposition to the scientific consensus and/or opposing action to prevent climate change). However, they also found that although Twitter discussions of climate change often occurred within polarizing “echo chambers,” they also took place within open forums, in which mixed-attitude communities of skeptics and activists often interact, thereby reducing polarization and stimulating debate. Pearce et al. (2014) also found high levels of polarization and echo chambers around climate change on Twitter, but identified mixed attitude communities too in the UK.

Finally, there is also evidence that “activist” Twitter users may have taken “the opportunities presented by the IPCC reports to defend climate science against attacks, whether or not the reports themselves are focused on the science” (Pearce et al. 2018, p. 6). A similar phenomenon has also been seen in the Twitter response to the three 2016 US presidential debates, where Twitter users used the debates as pegs to give air to their own concerns or agendas, rather than follow the main themes of debates, or in other words, the TV debates did not set the agenda for the Twitter debates (Zheng and Shahin 2018). This suggests that it is useful to look at the extent to which Twitter users responding to the SRCCL prioritized their own concerns and agendas beyond the report’s main findings and consequently design public facing communication efforts in ways that engage with such concerns more effectively.

## The IPCC and the communication of the SRCCL

The SRCCL was commissioned by governments in April 2016 as one of three special reports included in the IPCC’s Sixth Assessment Report cycle. The first of those reports published in October 2018 was the Special Report on the impacts of global warming of 1.5 °C above pre-industrial levels, known as SR1.5 (IPCC 2018). It stood out for its unequivocal statement that it would require rapid, far reaching, and unprecedented changes in all aspects of society to limit global warming to 1.5 °C. The SRCCL was a complementary report that came out the following year, and stressed that tackling land issues was part of the changes needed (IPCC 2019). The SRCCL calculated that agriculture, forestry, and other land use activities during 2007-2016 amounted to around 23% of total net anthropogenic emissions of GHG emissions.

The SRCCL’s full name is “Climate Change and Land, an IPCC special report on climate change, desertification, land degradation, sustainable land management, food security, and greenhouse gas fluxes in terrestrial ecosystems.” The variety of topics included in the title aptly illustrates the large number of themes it was asked to include. It was the first IPCC report in which a majority of the authors (53%) were from developing countries.^2^ As is customary with IPCC reports, the SRCCL did not include new science, but summarized existing knowledge, in this case gleaned from more than 7,000 cited references.

The formal launch of the Summary for Policy Makers (SPM)^3^ for the SRCCL took place in Geneva on 8 August 2019, with a press conference attended by the international media and several other journalists watching the live webinar and sending in questions.^4^ The IPCC press release stressed that finding a solution to the growing pressure on land use and food systems was only *part* of the solution for addressing climate change. Its opening paragraph highlighted that “keeping global warming to well below 2 °C can be achieved only by reducing greenhouse gas emissions from all sectors including land and food”.^5^ The press release formed one element of the IPCC’s outreach work prepared by the IPCC communications team and the lead authors, which also included the Headline Statements from the Summary for Policy Makers (SPM), FAQs, videos, and PowerPoint presentations.^6^ During the day of the launch, a regular stream of messages was posted on various social media platforms, particularly Twitter, Facebook, LinkedIn, and Instagram, of which Twitter was the most important.

Table 1 shows the presence or absence of twelve main topics gleaned from the different communication initiatives and platforms used by the IPCC. The topics, which are not mutually exclusive, are based on an inductive reading of (i) the main messages highlighted in the prepared statements by the chairs of the working groups in the opening 15 min of the press conference, (ii) the press release, and (iii) the headline statements from the SPM. As can be seen from the table, there was a high degree of consistency in the presence of these twelve topics found in these three communication channels. Illustrative quotes are included for each topic, with a record of one of the four sources for each of the topics (tweets, press release, SPM, or press conference).

**Table 1:** The main topics of the SRCCL by type of IPCC communication/platform

**Topics**	1. Summary key message: Land is under growing human pressure. Land is a part of the solution. But land cannot do it all	2. Climate change—land use interaction: How land use contributes to climate change and how climate change affects the land	3. Land degradation and desertification	4. Food security	5. Role of land critical in CO2 removal	6. Food waste
**Representative quotes**	“Land is where we live. Land is under growing human pressure. Land is a part of the solution. But land can’t do it all.” (tweet)	Agriculture, Forestry and Other Land Use activities represent<ing> 23% of total net anthropogenic emissions of GHGs; “Climate change creates additional stresses on land, exacerbating existing risks to livelihoods, biodiversity, human and ecosystem health, infrastructure, and food systems.” (SPM)	“When land is degraded, it becomes less productive, restricting what can be grown and reducing the soil’s ability to absorb carbon.” (press release)	“Food security will be increasingly affected by future #climatechange through yield declines, increased prices, reduced nutrient quality, and supply chain disruptions” (tweet)	“Limiting global warming to 1.5 or even 2 degrees will involve removing carbon dioxide from the atmosphere, and land has a critical role to play in its removal” (tweet)	The report records that about one-third of food produced is lost or wasted. <..>Reducing this loss and waste would reduce greenhouse gas emissions and improve food security. (press release)
Press release	**✔**	✔	✔	✔	✔	✔
Press conference	**✔**	**✔**	**✔**	**✔**	**✔**	**✔**
Headline statements from the SPM	✖	**✔**	**✔**	**✔**	**✔**	**✔**
IPCC tweets	**✔**	✔	✖	**✔**	✔	✔
Topic	7. Increase in land surface temperature	8. Indigenous knowledge	9. Variety of solutions needed	10. Balanced diets, different diets, less meat	11. Land management/diversification/sustainability	12. Early/urgent action needed
Representative quotes	“Temperatures over land surfaces have increased by almost twice the global average.” (press conference)	“Indigenous and local knowledge can contribute to overcoming the combined challenges of climate change, biodiversity loss, food security, desertification and land degradation” (press conference)	“There are solutions in the hands of farmers. But there are also solutions in the hands of each of us, when we buy food, and don’t waste food.” (tweet)	“Balanced diets featuring plant-based foods, such as coarse grains, legumes, fruits and vegetables, and animal-sourced food produced sustainably in low greenhouse gas emission systems, present major opportunities for adaptation to and limiting climate change.” (press release)	“We are best placed to tackle #climatechange in a world with an overall focus on sustainability” (tweet); “Policies that support sustainable land management, ensure the supply of food for vulnerable populations, and keep carbon in the ground <..> are important.” (press release)	“Urgent action, and mid-term action, would take us into a better future” (tweet)
Press release	✖	✖	✔	✔	✔	✔
Press conference	✔	**✔**	**✔**	**✔**	**✔**	**✔**
Headline statements from the SPM	**✔**	**✔**	**✔**	**✔**	**✔**	**✔**
IPCC tweets	✖	✖	✔	✔	**✔**	✔

We have omitted some topics from Table 1 (such as regional differences, biodiversity, extreme weather events, and the number of authors from developing countries) or included them under one broad heading (for example, land management/diversification/sustainability). This was done partly to reduce the number of topics into the twelve that were most commonly found, but also because some topics were often lumped together in the messaging.

Only the presence (or absence) of topics was assessed, and not the salience (measured by headlines or early mentions), or dominance (overall weighting of topics) (cf. Painter 2013, p. 58). One simple measure of the relative dominance of topics can be achieved by a word search in the press release. This shows that “food security” appears nine times, the same number as “degradation” and “sustain*”; “Land management,” “food waste,” and “desertification” five times; and “early action” and “diet*” three times.

Table 2 shows a selection of the tweets posted by the IPCC on Twitter on the day of the launch, taken from the Twitter data set described in Section 4 below and from the list of tweets the IPCC posted that day. The table only shows those tweets which highlighted elements of the content of the report consistent with the messages found in the press conference and press release and does not include other tweets which were designed to draw attention to the release of the report. As can be seen, the tweets largely reflect the topics and language identified in Table 1 and particularly topics 1, 2, 4, 5, 9, 10, and 12. There were two official tweets on 8 August about dietary changes (topic 10), the first made in the name of Debra Roberts, the co-chair of IPCC WG2, and the second by Jim Skea, the co-chair of WG3.

**Table 2:** IPCC tweets on 8 August 2019

1. #IPCC Special Report on #ClimateChange and Land: Land is where we live. Land is under growing human pressure. Land is a part of the solution. But land can’t do it all. #SRCCL press release	
2. Urgent action, and mid-term action, would take us into a better future—Hans-Otto Pörtner, co-chair of #IPCC Working Group II #SRCCL #ClimateChange #GlobalGoals	
3. Food security will be increasingly affected by future #climatechange through yield declines, increased prices, reduced nutrient quality, and supply chain disruptions—Priyadarshi Shukla, co-chair of #IPCC	
4. Climate change will exacerbate warming trends that already exist in cities—Debra Roberts, co-chair of #IPCC Working Group II #SRCCL #GlobalGoals	
5. We are best places to tackle #climatechange in a world with an overall focus on sustainability—Hans-Otto Pörtner, co-chair of #IPCC Working Group II #SRCCL #GlobalGoalspic.twitter.com/uFOUHYAOEb	
6. Land is a critical resource – we rely on it for food, water, health and wellbeing – but it is already under growing human pressure. #ClimateChange is adding to these pressures. #SRCCL #IPCC #GlobalGoals	
7. Limiting global warming to 1.5 or even 2 degrees will involve removing carbon dioxide from the atmosphere, and land has a critical role to play in its removal—Jim Skea, co-chair of #IPCC Working Group III #SRCCL #ClimateChange #GlobalGoalspic.twitter.com/m34S8CUaqo	
8. There are solutions in the hands of farmers. But there are also solutions in the hands of each of us, when we buy food, and don’t waste food—Valérie Masson-Delmotte, co-chair of #IPCC WG I #SRCCL	
9.This is the first time in IPCC report history that a majority of authors – 53% – are from developing countries, says IPCC Chair Hoesung Lee #SRCCL #ClimateChange #GlobalGoalspic.twitter.com/UbgrFCl9Z7	
10. IPCC does not recommend people’s diets. What we’ve pointed out on the basis of the scientific evidence is that there are certain diets that have a lower carbon footprint—Jim Skea, co-chair of #IPCC WGIII #SRCCL	
11. Balanced diets featuring plant-based foods, such as coarse grains, legumes, fruits and vegetables, and animal-sourced food produced sustainably in low GHG emission systems, present major opportunities for adaptation to and limiting climate change—Debra Roberts, co-chair of IPCC Working Group II	
12. There are things we are already doing. We are using technologies and good practices, but they do need to be scaled up and used in other suitable places that they are not being used in now—Panmao Zhai, co-chair of IPCC Working Group I	
13. Food security will be increasingly affected by future climate change through yield declines – especially in the tropics – increased prices, reduced nutrient quality, and supply chain disruptions—Priyadarshi Shukla, co-chair of IPCC Working Group III	
14. Agriculture, forestry and other types of land use account for 23% of human GHG emissions. At the same time natural land processes absorb CO2 equivalent to almost a third of CO2 emissions from fossil fuels and industry—Jim Skea, co-chair of IPCC Working Group III	
15. New knowledge shows an increase in risks from dryland water scarcity, fire damage, permafrost degradation and food system instability, even for global warming of around 1.5°C—Valérie Masson-Delmotte, co-chair of IPCC Working Group I	
16. Land already in use could feed the world in a changing climate and provide biomass for renewable energy, but early, far-reaching action across several areas is required. Also for the conservation and restoration of ecosystems and biodiversity—Hans-Otto Pörtner, co-chair of IPCC Working Group II	

As the SPM made clear, diet and dietary change was only part of the array of solutions discussed.^7^ In the press conference, the speakers were keen to stress that there was no “single silver bullet,” but 40 policy solutions across land types, bioclimatic regions, or local food systems (IPCC 2019, chapter 6).

However, during the conference, there were several questions about dietary options as a solution. Jim Skea, the co-chair of WG3, summarized the position of the IPCC, stressing that the IPCC in general does not make recommendations but provides evidence for policy makers; in the SRCCL, it was not making specific recommendations on diets and that dietary choices were often shaped by local production practices, factors, and cultural habits.^8^

In summary, the key point here is that the IPCC’s communication efforts stressed that the SRCCL presented a menu of land changes to help tackle climate change, of which dietary changes formed a relatively small part. This was reflected in several summaries or briefings published around the time of the publication of the report by specialist organizations.^9^

## Research questions, data, and methods

### Research questions

Given the context outlined above, we were interested in analyzing the response by Twitter users to the SRCCL, with a particular focus on identifying the dominant topics prompted by, or linked to, the Report’s release (RQ1) and their relative prevalence (RQ2). In addition, we wanted to explore the extent to which these topics differed from the IPCC-promoted messages outlined in Section 3(RQ3) and identify in which of these topics the level of contention was highest as measured by sentiment polarity and toxicity (RQ4).

### Data

We used two tools for collecting relevant tweets during the month of August 2019: TAGS,^10^ a software that automatically collects and archives tweets matching a given query sent to the Twitter API with a historical search range of 7 days, and TwitterScraper,^11^ a Python library that collects tweets based on a given query but without a limit on historical search range. Both of these methods search only a subset of full Twitter activity due to Twitter’s data collection restrictions. We used the keyword “ipcc” to collect the widest sample possible.

The resulting dataset consisted of 27,259 tweets in 41 languages. We used another Python package called langdetect to detect the language of each tweet.^12^ There were 17,933 English tweets in our dataset. Considering the prevalence of English tweets as well as the fact that most natural language processing implementations are best trained and validated on English language corpora, we focus on this subset of tweets in this paper.

Within this subset of the data, there was a large number of verbatim retweets (i.e., exact copies of the original tweet), irrelevant or spam tweets, and several tweets related to another IPCC report (on the Ocean and Cryosphere). We also investigated the dataset for the presence of bots by analyzing the activity distribution across users. Bots are known to have high output but low follower/following counts. We removed the tweets of accounts following this pattern, as suggested in Jang and Hart (2015). After removing as many verbatim retweets, irrelevant, and bot tweets from the dataset as possible, we were left with 6,441 “valid,” i.e., relevant English tweets. We removed verbatim retweets from the dataset as we did not want to include a frequency bias in our topic model. We were interested in the content of original tweets, not in endorsements of those tweets, manifested primarily via retweet. Duplicates of the same text as input in our model would encourage the algorithm to over-represent the semantic context of these duplicates and therefore bias the resulting topic construction.

### Methods

To address the RQs, we applied a combination of quantitative and qualitative methods to the described data (for a full description of our methods, and in particular CorEx (correlation explanation), please see the Supplementary Material). To analyze the content of the tweets, we started by topic modelling, which is an unsupervised machine learning paradigm that normally relies on Bayesian probability to determine the underlying thematic structure of a corpus of text input (Lafferty and Blei 2007). Critically, topic models do not require prior labelling of the documents to be classified; the thematic structure emerges from the generative topic model (Blei 2012). It has become particularly useful for analyzing large corpora of text that could not be realistically and reliably analyzed by humans. Successful examples of topic modelling application in policy research include studies on online petitions to the UK Government (Vidgen and Yasseri 2020), the European Refugee Crisis (Heidenreich et al. 2019), and national cyber security strategies (Kolini and Janczewski 2017). Specifically related to climate, Dahal et al. (2019) use topic modelling and sentiment analysis to identify and analyze themes in the general climate change Twitter discourse. While topic models do not provide external validation of the results they produce, they are very useful for generating an overview of a large input dataset and providing insights into its thematic structure. However, to add domain knowledge we fed the model with “anchors,” selected based on the assumption that in addition to the general content of the SRCCL around food and land systems outlined in Section 3 and the dominant sources of controversy in previous IPCC report discourses outlined in Section 2.2 (i.e., general criticism of the IPCC’s scientific credibility and/or skepticism of the results discussed), the discourse around the SRCCL report would also include a new topic around meat eating and diet (see Section 3).

Then, we calculated the values of sentiment polarity and toxicity for each tweet separately because of their ability to help us detect contention in the discourse. Sentiment polarity refers to how negative, positive, or neutral the overall sentiment of a given piece of text is. The toxicity measure of a text reflects the amount of rude, disrespectful, or insulting language it contains. Previous work has demonstrated the connection between toxicity, negative sentiment, and contention in various discourses on Twitter. Contention may be characterized as speech intended to voice disagreement, criticism, or dissent and includes language ranging from innocuous disagreement to hate speech and “trolling,” i.e., insulting antisocial posts. Salminen et al. (2020) use toxicity and sentiment to detect hate speech, while Cheng et al. (2017) find toxicity in particular to be a strong predictor of trolling behavior on social media.

Thus, we suggest that topics in our model with higher values of toxicity and more negative sentiment would imply higher levels of contention within these topics, which we could then manually evaluate by inspecting specific tweets. To calculate sentiment polarity and toxicity, we used the VADER sentiment analysis tool and the Perspective API, respectively. In the VADER analysis implementation, sentiment scores range from -1 to 1, with more negative scores indicating more negative sentiment (vice versa for positive scores) and values around zero indicating neutral sentiment. Toxicity values using perspective range from 0 to 1, with values closer to zero indicating lower toxicity and values closer to 1 indicating higher toxicity. For more details of the calculations of sentiment polarity and toxicity levels, please see Supplementary Material.

## Results

### Topic distribution and levels of contention

Table 3 summarizes the results of the anchored topic model with the five topics distinguished as Diet and Consumption (Topic 1), the Report Publication (Topic 2), Land Use and Food Systems (Topic 3), Climate Skepticism and Criticism of the IPCC (Topic 4), and Miscellaneous (Topic 5). The second row of the table lists the key words under each topic. Looking at the third row, we can see how many tweets were assigned to each topic, thereby reflecting the relative prevalence of each topic in the sample. As can be seen, Topics 2 (Report Publication) and 3 (Land Use) were the most commonly found by some margin. However, the number of tweets assigned to Topic 1 (“Diet”) is also high (895 tweets) and higher than the number of tweets assigned to Topic 4 (Skepticism).

**Table 3:** Distribution of words in Topics with “anchoring”

	Topic 1: ***Diet and Consumption***	Topic 2: ***Publication of Report***	Topic 3: ***Land Use and Food Systems***	Topic 4: ***Climate Skepticism and Criticism of IPCC***	Topic 5: ***Miscellaneous***
Keywords	meat, consumption, diet, vegan, diets, dairy, eating, plant, animal, reduce, foods, eat, balanced, waste, plantbased	report, new, special, latest, released, today, shows, read, week, highlights, leaked, summary	land, food, use, change, srccl, climate, security, sustainable, production, pressure, degradation, way, management, human	science, fake, tech, hoax, bcpoli, cdnpoli, peer, showing, impact investing, leadership, solar, fraud,scam,fact,puppet	geneva, chair, plenary, working, authors, session, group, present, meeting, consider, development, conference, author, opportunities
Number of tweets	895	3,102	3,213	851	1,679
Toxicity	0.138 (0.0967)	0.0921 (0.0719)	0.0956 (0.0740)	0.180 (0.111)	0.104 (0.0732)
Sentiment polarity	0.105 (0.0958)	0.172 (0.296)	0.146 (0.226)	0.0468 (0.0)	0.182 (0.273)

Rows 4 and 5 show the toxicity and sentiment polarity values of each topic, first the average then the standard deviation in parenthesis. Toxicity values range from 0 to 1, with values closer to zero indicating lower toxicity and values closer to 1 indicating higher toxicity. Sentiment scores range from -1 to 1, with more negative scores indicating more negative sentiment (vice versa for positive scores), and values around zero indicating neutral sentiment (see Supplementary Material). High toxicity scores tend to be associated with tweets from individuals. Such tweets found in our sample include strong swear words, personal abuse often questioning the original poster’s intelligence (if posted in response to another tweet), and high levels of other forms of incivility.

To illustrate this point, two examples are given of paraphrased tweets from the Diet Topic with high toxicity and negative sentiment. We include the sentiment and toxicity scores of the original tweets in parentheses. The first one refers to a question asked to the IPCC panel during the launch press conference:

“BBC reporter asks if the panel has reduced meat eating!!!! Fuck wits ... #ipcc #questions” (Sentiment: -0.4, Toxicity: 0.93)

The second example was posted by an activist claiming that because IPCC did not explicitly recommend vegan diets, their environmental concerns are inauthentic.

“About #IPCC ... We're #Vegan! It means SAVE #ANIMALS and SAVE THE #EARTH! If you're not Vegan, please shut up your f*cking mouth about your concern IPCC. Poser!” (Sentiment: -0.75, Toxicity: 0.96)

The key result here is that the Diet and Skepticism Topics appear on average more toxic and also more inclined towards negative sentiment than the other Topics. Further inspection of the tweets assigned to these Topics indicates the extent of the controversy. Within the Diet Topic, we see similarly high levels of toxicity and negative sentiment from both the two antagonistic groups—what we can call broadly the meat/dairy supporters and the plant-based advocates. We detect two extremes: one group of people criticizing the IPCC for, in their view, slandering the meat and dairy industries, and trying to take away their right to eat meat, and another group of people, apparently vegans and vegetarians, criticizing meat eaters for contributing to climate change. Often these tweets, from both camps, exhibit violent and toxic language aimed at the antagonists they respectively identify: the IPCC, its scientists, and supporters for the first camp, or meat and dairy consumers for the second. We observe antagonism against various elements of the media in both camps.

For example, the meat/dairy supporters in our sample tend to perpetuate the simplified narrative that the IPCC is “scaremongering” by claiming that meat-based diets cause climate change. Others in this camp use the idea of the IPCC advocating plant-based diets to invalidate the science of climate change in general and hold up the SRCCL as evidence of the IPCC’s incompetence. Moreover, some argue that the meat and dairy industries actually reduce carbon and that eating meat saves the planet. Some meat/dairy supporters also ridicule the media for apparently supporting the IPCC and the SRCCL. On the other hand, extreme plant-based advocates proclaim that anyone who does not listen to the IPCC and adopt more vegetarian/vegan choices or plant-based diets actively contributes to climate change and thus should suffer severe consequences and/or feel ashamed. Interestingly, we also observe individuals who are vegan but also antagonistic towards the IPCC for not making more explicit recommendations for plant-based diets. Finally, these advocates identify elements of the media succumbing to the pro-meat narrative and find fault in them for propagating it.

We also observe a bulk of tweets in this Topic coming from the official IPCC account as well as the accounts of the report’s authors and supporters which aim explicitly to emphasize the absence of an official recommendation to adopt plant-based diets in the report (see Section 3 and Table 2). These tweets exhibit very low toxicity and are generally neutral in terms of sentiment polarity. The presence of these tweets in the Diet Topic might help to explain its lower average toxicity level than that of the Skepticism Topic, despite the high toxicity level of many of the other tweets.

For example, a tweet from a climate activist in favor of the SRCCL findings demonstrates the effort to combat the perception that the IPCC was trying to urge plant-based options on the public with positive, yet calm, language. A paraphrase of the tweet is as follows:^13^

“We would not recommend to people what they should consume. But it would help the climate and the health of humans if consumers in many developed countries ate less meat, and if politicians gave incentives to encourage such behaviour ”#ClimateAction #SRCCL #IPCC”

The tweet has a high positive sentiment polarity score of 0.938 and a relatively low toxicity score of 0.106. It was assigned to the Diet Topic, which substantiates our claim that tweets such as this one bring down the average toxicity value for this topic despite the very high outliers.

Upon further examination of the Skepticism Topic, we observe the highest toxicity level of all the topics. Given the history of skepticism and hostility directed towards the IPCC discussed in Section 2.2 and the accusations of the organization simply being used as a “puppet” of the UN to push a political agenda, we anticipated a certain degree of this toxicity. However, we suspect that these levels could be higher than those observed in the discourse surrounding previous reports due to the incorrect perception that the IPCC was “attacking” the meat and dairy industries.

We again see two factions contributing to the overall high toxicity of this topic: those attacking the IPCC (generally climate change deniers) and those refuting those attacks (generally climate activists). Both display more or less equal levels of toxic language.

For example, a paraphrase of a tweet posted by another climate activist is as follows:

“Even if we gave you thousands of facts about the settled science of climate change, you would still be in denial. Read scientists like Arrhenius, Tyndall and others. 20,000 IPCC scientists say you are an IDIOT. #climatechange #gretathunberg #rechauffementclimatique.”

The original tweet has a negative sentiment polarity score of -0.746 and a high toxicity score of 0.767. It has been assigned to the Skepticism Topic and pertains mostly to refuting the skeptics who object to the IPCC on the basis of its scientific validity with rather harsh words and a negative attitude.

The other topics, particularly the Report Publication and Land Use Topics, exhibit generally low toxicity and neutral to higher sentiment polarity. In the case of the Report Publication Topic, which we identified as pertaining primarily to spreading awareness of the report, we see significantly higher sentiment polarity than the base model and corpus average. A majority of the tweets assigned to this Topic focus simply on calling attention to the report and hinting at its major findings. For the Land Use Topic, identified as representing further discussion on the findings related to details of the implications for climate change and land use of the global food production system, we observe low toxicity and neutral sentiment. We suspect this could be due in part to the relative complexity of the language used in these tweets, and their focus on arguably more complex subject matters relating to the underlying science. The key words identified in this Topic listed in row 2 of Table 3, such as “degradation,” “sustainable,” “pressure,” “security,” and “management” to a notable extent reflect the main topics identified in the official IPCC messaging around the SRCCL. However, these concepts may be less immediately understandable by a non-specialist audience and thus perhaps a less important driver of public interactions on the subject.

### Links to URLs

Research shows that links to URLs included in Tweets which point to related news are important tools for popularizing and promoting messages more powerfully than the narrative in its text-only form (Blanquart and Cook 2013) and thus for increasing virality via retweets (Suh et al. 2010). We also know that reports on mainstream media act as an important prompt for social media discussions and contention both in general about climate change (Pearce et al. 2014; Grouverman et al. 2019) and in particular about IPCC reports (Newman 2017). For example, analysis of large Twitter data sets from 2012 to 2014 shows that the contention around climate change on tweets generally mirrors much of the controversy observed in traditional media (Jang and Hart 2015). Grouverman et al. (2019) found that “professional news outlets” (which included established and new media outlets) represented 33% of the types of news and information sources shared by Twitter users discussing climate change. In a similar fashion, Newman (2017) found that “mainstream media,” which he defined as referring to media outlets such as *CNN* or *The Washington Post*, represented the most important source of information measured by the hyperlinks Twitter users embedded in their tweets (35% of all URLs to external websites).

In our sample, the overwhelming majority of the tweets (87%) had links attached to URLs. These URLs included the IPCC report itself or IPCC posts, a variety of pictures, individual Twitter accounts, YouTube videos, climate/food/energy niche sites, and the sites of interest and advocacy groups. For example, links to the in-depth report by the climate specialist site, Carbon Brief, on the SRCCL appeared 20 times^14^, while a variety of YouTube videos were linked to 19 times. An examination of a random sample of around 500 tweets would suggest that about 10% included links to the URLs of articles in mainstream media organizations.

Table 4 shows that of the mainstream media articles included in tweets, *The Guardian* had by far the most links (154), followed by the *BBC* (52). It is notable that five UK-based media organizations whose websites are amongst the most used and trusted (Newman et al. 2020, p. 62) led their coverage on the SRCCL with a focus on eating less meat in their headlines. As Table 5 shows, these were the *BBC*, *The Telegraph*, *The Mail*, *The Times*, and *The Independent*. The London-based news agency *Reuters* too led one of its main articles on the SRCCL with the headline “U.N. flags need to cut meat to curb land use impact on global warming.”

**Table 4:** Number of links to URLs by news media organization*

Media outlet	Number of links
Guardian	154
BBC	52
The Atlantic	41
New York Times	30
CNN	16
Vox	15
Independent	13
Wired	13
ABC (Australia)	12
Irish Times	8
National Geographic	7
Time Magazine	5
Telegraph	4
Vice	3
Wall Street Journal	3
Washington Post	3
Huffington Post	3

*Links to articles in The Conversation and Nature also appeared more than 30 times, but are not included here due to their not being regarded as examples of mainstream news media

**Table 5:** Media coverage of the SRCCL, on 8 August 2019

**Source**	**Headline**	**Text after headline**
Reuters	U.N. flags need to cut meat to curb land use impact on global warming	Global meat consumption must fall to curb global warming, reduce growing strains on land and water and improve food security, health and biodiversity, a United Nations report on the effects of climate change concluded.
Associated Press	UN climate report: Change land use to avoid a hungry future	Human-caused climate change is dramatically degrading the Earth's land and the way people use the land is making global warming worse, a new United Nations scientific report says.
Agence France Presse	Land use overhaul vital for food and climate security: UN	The world must overhaul how it feeds itself and manages Earth's forests, pasture and resources in order to avoid food insecurity and the worst impacts of climate change, a landmark UN assessment said Thursday.
New York Times	Climate Change Threatens the World’s Food Supply, United Nations Warns	The world’s land and water resources are being exploited at “unprecedented rates,” a new United Nations report warns, which combined with climate change is putting dire pressure on the ability of humanity to feed itself
Washington Post	Changing climate imperils global food and water supplies, new U.N. study finds	Agriculture and other land use accounts for 23 percent of human-caused greenhouse gas emissions.
Los Angeles Times	Land use policy key to reining in global warming, U.N. report warns	Slashing greenhouse gas emissions from cars and power plants won’t be enough to avoid the worst effects of climate change.
USA Today	UN report: Climate change threatens our food supply. Here's how we can fix it	Global warming on land is happening at a rapid rate, and humans will need to change the way they eat and farm to help save the planet, a new United Nations report says.
Fox News (online)	UN climate report: Change land use to avoid a hungry future (Associated Press)	Human-caused climate change is dramatically degrading the Earth's land and the way people use the land is making global warming worse, a new United Nations scientific report says.
CNN (online)	Change food production and stop abusing land, major climate report warns	Humans have damaged around a quarter of ice-free land on Earth, United Nations scientists warned in a major report Thursday, stressing that further degradation must be stopped to prevent catastrophic global warming.
Guardian	Climate crisis reducing land’s ability to sustain humanity, says IPCC	UN report finds ecosystems never before under such threat and restoration is urgent
Telegraph	People should adopt a plant-based diet to prevent climate change but British beef farmers call report 'misleading'	A new UN report has suggested people take up plant-based diets to prevent climate change.
Times	Eat less meat to save the Earth, urges UN	Cutting food waste and eating less meat will reduce climate change by saving millions of square miles of land from being degraded by farming, according to a United Nations report.
Mail	Eating less meat could help save the planet by preserving MILLIONS of miles of land from farming and cutting greenhouse gases, UN report finds	Experts from across the globe met to discuss the findings in Geneva this week
BBC	Plant-based diet can fight climate change – UN	Switching to a plant-based diet can help fight climate change, UN experts have said.

The *BBC*’s article on 8 August was headlined ‘Plant-based diet can fight climate change – UN’, and was linked to 19 times in our sample. It was shared on Twitter 5,000 times and attracted over 3,000 comments. The article quoted one of the authors of the SRCCL, Professor Pete Smith from Aberdeen University in the UK, as saying that “We’re not telling people to stop eating meat. In some places people have no other choice. But it’s obvious that in the West we're eating far too much.”

However, there is a mixed picture as to how much attention the mainstream media paid the dietary issue, and therefore how much influence it may have exerted. First, *The Guardian*’s main article on the SRCCL on 8 August did not lead with the dietary question, although it was discussed lower down in the piece.^15^ This article appeared in tweets on fifty occasions. Secondly, of the main news agencies, *Reuters* did lead on this aspect, but *AP* and *AFP* did not. Moreover, some of the UK titles listed in Table 5 hardly featured in the Tweet attachments to URLs. And in the USA, although *Time* magazine, the *Wall Street Journal*, *Vice*, and *Vox* did lead on some aspects of meat eating or the meat industry, several major titles such as the *New York Times*, *Washington Post*, *USA Today*, *LA Times*, *Fox News*, and *CNN*’s first published article on the SRCCL did not lead on the need to eat less meat, but instead focused on the impact of climate change on the world’s food supply (see Table 5).

The emphasis by the IPCC on not making policy recommendations (about diet) was picked up by several of the media organizations, including the *BBC* and the *Telegraph*. However, these qualifications were not enough to prevent the main industry organization representing farmers in the UK, the National Farmers’ Union (NFU), from accusing media organizations of bias for wrongly (in their view) focusing on the dietary aspects of the SRCCL, an emphasis which the NFU described as “incredibly frustrating”.^16^ The priority given by some of the media to the dietary question was also highlighted by the prominent climate policy skeptic, Bjorn Lomborg^17^, and was also pointed out by other, non-skeptical, sources (Sauer 2019; Black 2019).

## Discussion

The prevalence of Topic 2 (Report Publication) in our results chimes with existing research on common topics found in Twitter responses to previous IPCC reports. Newman (2017) found tweets that were simply relating news and information to a wider audience, and giving information about access to key IPCC events (which he labelled ‘public understanding’), represented the highest percentage, namely, 27% of the 100 most propagated tweets he analyzed in response to the IPCC’s AR5. Similarly, his categories of “settled science” (mostly referring to greater certainty about the human causes of climate change), climate “impacts and risks,” and “technology,” which captured the content of the AR5, together amounted to 30% of the tweets. Therefore, it should also not be surprising that the Land Use Topic is the most common topic found in our sample, particularly as we saw in Section 3 that firstly, there were twelve different common topics in the official message around the SRCCL (Table 1), and that most of these were found in the official IPCC tweets posted on 8 August (Table 2).

However, in contrast, the prevalence of the Diet Topic is much higher than what one would expect. First, as set out in Section 3, Diet and Consumption was just one of the twelve topics in the official messaging and was not a primary message of the report, and secondly, it featured in only two of the sixteen tweets officially posted. Our results suggest that while the SRCCL did not make any explicit recommendation on diet change as a solution for climate change, the issue emerged as a source of contention in the discourse surrounding the IPCC and their scientific work. As discussed above, in previous social media responses to IPCC reports, the main point of contention in the Twitter discourse has pertained to skepticism about the authenticity of the IPCC’s work and its scientific authority (Pearce et al. 2014; Jang and Hart 2015; Dahal et al. 2019). In the response to the SRCCL however, the perception of the IPCC advocating a shift away from meat-based diets generated a new topic and controversy.

It is difficult to ascertain the exact extent of the role that the mainstream media played in shaping the ways the SRCCL was discussed on Twitter. Journalistic norms of novelty and controversy and the imperative to make a complicated report relevant to audiences in the developed world were two of the factors which resulted in several media organizations headlining one aspect of the SRCCL at the expense of others, in the process prompting more social media discourse on this topic which may not have happened to the same extent without such prompts. However, as we saw in Section 5.2, several other prominent media organizations led on changing land use and climate change impacts, and such articles were often embedded as a hyperlink in tweets. Also, links to mainstream media only made up an estimated 10% of the total number of links in the tweets. Finally, we are assuming in line with previous research that links promote message content and virality (Suh et al. 2010; Blanquart and Cook 2013), but we do not know from our sample whether users actually clicked on the links.

It is also the case that in our case study Twitter users clearly followed their own agendas, as previous research has shown. As discussed above, Zheng and Shahin (2018) found that users in the USA had their priorities as to what they wanted to discuss around “media events,” independently of the prompts coming from those events. Their analysis of around 300,000 tweets found that none of the main issues raised during the televised presidential debates (the Syrian civil war, terrorism, Russia, immigration, job creation, and Trump’s taxes) were extensively discussed on Twitter; instead, the conversations were mostly about candidate-specific issues. Cherry-picking some areas of a scientific report would also be consistent with previous findings that Twitter users seize opportunities presented by IPCC reports on climate change to promote their own views beyond the focus of such reports (Pearce et al. 2018).

It is clear from our results that the topic of meat eating, dietary change, and animal agriculture was both a major topic of conversation and of contention. Previous research has mapped patterns of incivility, sarcasm, or rudeness on Twitter around climate change, found also in our analysis of tweets. Here we are not arguing that incivility is necessarily an inappropriate or illegitimate reaction to the meat and dietary question in particular, or the IPCC report in general. Emotional reactions to contentious issues, particularly when underpinned or provoked by anger, have shown to be an important and understandable response to gender or racial injustice (e.g., Lorde 1997), and to climate threats (du Bray et al. 2019), and are to be expected given the nature of public responses on Twitter. However, as mentioned earlier, analysis of tweets in the USA showed that patterns of sarcasm and incivility were much more common amongst those holding skeptical perspectives on climate change and right-leaning politics, while civil tweets were significantly more likely to be associated with profiles indicating an affinity with left-wing political views (Anderson and Huntingdon 2017). In contrast, our results suggest that some pro-vegan or vegetarian discourses (often seen as coming from a more left-leaning viewpoint) can be just as rude or impolite as those defending meat eating and the meat industry.

## Conclusions

In answer to our research questions outlined in Section 4.1, the CorEx method gives robust results identifying the five most common topics found in the Twitter responses to the SRCCL. These centered on the public messaging of the report, the content of the report around land issues, dietary choices, and the credibility of the IPCC and its science (RQ1). While it was to be expected that the first two of these featured prominently, the stand-out result was the amount of discussion around meat eating and the meat industry, which was the third most discussed topic (RQ2). This was not in line with the dominant messaging from the IPCC which focused on land options and solutions to tackle climate change and not so much on dietary options (RQ3). Twitter users brought their own agendas to the discussion of these issues beyond the main content and messaging from the report which prompted them. Finally, we found high levels of contention, measured by toxicity and sentiment polarity, around the topics of dietary choices and the credibility of the IPCC (RQ4). Tweets from both those defending meat eating and from those attacking them included similar amounts of violent and toxic language aimed at their antagonists.

As outlined in Section 2, there is much more published research on social media discourses on climate change/science and the IPCC than on the nexus between diet, animal agriculture, and climate change. This study helps to fill the gap, with our two most important conclusions being that as follows:

- Meat reduction policies and the role of animal agriculture in GHGs have strongly entered the climate change arena as a new source of contention and in response to IPCC reports in particular;
- The existing polarization of the meat debate on social media (as evidenced for example by the response to the EAT-Lancet report earlier in 2019) strongly shaped the Twitter responses to the SRCCL and could have been anticipated more effectively;

In addition, although we did not carry out a comprehensive quantitative content analysis of the different types of pro or anti-meat narratives found in our sample, it is clear from our description of them in Section 5.1 that they exhibit a multiplicity of narratives (cf. Maye et al. 2021). Both sides commonly seek to legitimize their existing positions by siding with or against the IPCC, either criticizing it for scaremongering or for (wrongly in their view) making the link to climate change, or appealing to its authority to justify attacks on those who eat meat. However, the range of narratives includes anti-meat advocates who criticize the IPCC for not being more explicitly in favor of plant-based diets. Further research could illuminate the details and nuances of these meat narratives and counter-narratives both on Twitter and on other social media platforms having a wider demographic than Twitter, and the way different Twitter users bundle together pro-meat or anti-meat attitudes with other cultural identities.

Our results also support the view that climate controversies may have partly shifted from the direct denialism of human-induced climate change to the rejection of the science behind various solutions such as meat production and consumption (Schmid-Petri et al. 2017; Olausson 2019). Analysis of 40 YouTube videos posted in response to the SR1.5 showed that the discussion of policy options and solutions was the most prominent as a recurrent theme (Bounegru et al. 2020). This marked a sharp contrast to its lower prominence in previous studies such as O’Neill et al. (2015), who looked at the dominant frame responses in mainstream and social media to the AR5. Some scientists (e.g. Mann 2021) argue that the new type of contention around policy options is part of a well-funded strategy by climate denialists who used to dispute climate change, but who now try to block the action needed to move towards a low-carbon future (climate obstructionism). The controversy over meat eating in the climate change policy debate can be seen as one manifestation of a new “culture war,” as right-wing media in the USA and UK responded to President Biden’s announcement in April 2021 of deep cuts in US GHG emissions by claiming falsely that he was trying to stop Americans eating meat.^18^

Our findings have important implications for the communication of IPCC reports. First, the response on Twitter to the SRCCL has parallels with the response by mainstream and social media to the IPCC’s SR1.5 report of October 2018. Studies have shown that in this case, numerous articles chose to cite 2030 as a deadline for action which was a headline-driven interpretation of the IPCC’s statement that global emissions had to be reduced by 45% from 2010 levels by 2030 in order to avoid passing 1.5 °C (Boykoff and Pearman 2019). The so-called “12-year deadline” narrative became a clarion call for some politicians and climate activist groups like Extinction Rebellion to urge rapid and radical action.

The IPCC was fully aware in the communication both of the SR1.5 and SRCCL of the way the mainstream media were focusing on certain aspects of the reports and in the launch press conferences pushed back against either the focus (SRCCL) or the interpretation (SR1.5). Several IPCC authors involved with the SR1.5 chose to clarify publicly that the IPCC report did not say there were 12 years left to save the world^19^ and to stress the dangers of such a narrative, but the IPCC did not comment officially in response to this narrative, a silence which some authors criticized for being a “communication vacuum” (Raman and Pearce 2020) or for following a “safe option” that allows the IPCC to “retreat into a comfort zone that appears to preserve its integrity as a policy-neutral advisor” (Asayama et al. 2019, p. 571). Nor was there an official IPCC comment on the “dietary prioritization,” even though again individual authors did respond.

So the IPCC needs to address how it can respond more effectively to distortions of the content of its reports. Although it has been hampered in the past by the lack of capacity and resources amongst the IPCC communication team to engage in sustained “corrective” discussion on social (and mainstream) media, it seems important to re-evaluate the policy, particularly in a context where parts of the media will focus on short, easily digestible messages in preference to the complexity and nuance characteristic of SPMs.

Secondly, the scientists and communicators involved with the development and dissemination of IPCC reports need to deepen their understanding of how landmark, science-based reports are communicated and discussed on social media. As Garcia et al. (2019) showed, advocacy and campaign groups were very successful in organizing a digital countermovement in anticipation of the EAT-Lancet report using Twitter’s #feature (Jackson et al., 2020) and may have swayed undecided Twitter users. In the case of the SRCCL, there is little evidence of an orchestrated response on Twitter (and no specific hashtag in opposition to the report), but rather a group of individuals and groups tweeted their hostility to plant-based diets and climate-diet science. However, an already existing polarized debate about meat eating was transferred into the domain of responses to a report. It was almost inevitable that these public narratives would distort the key findings of the SRCCL and create a negative backlash. Perhaps most importantly, they could have been anticipated.

Although the IPCC has travelled a long way since its early days by allocating more professional resources to the communication of its reports and adapting to an age of online communication and social media (Hickman 2015; Lynn 2018), it is yet to come to terms fully with the ways digital technology alters and shapes the public discussion of their reports in ways that make climate solutions in particular more contested and more politicized and will do so in the future. In this context, as argued by Garcia et al. (2019), scientific organizations need to understand much better how social media are an integral part of the conversation about science, and although scientific communication on these platforms is much more complex than through traditional mainstream media, tools and techniques exist to capture and analyze public conversations to tackle that complexity when talking to the wider publics.^20^ Understanding advocacy and disinformation campaigns on social media (Benkler et al. 2018) and anticipation of likely (negative) responses based on prior analysis of the polarizing nature of the public policy issues (Bright et al. 2014) should be part of the approach. The next round of the IPCC’s Expert Communication Panel should address how the IPCC can best do this within tight time and budgetary constraints and within the constraints of its current policy-neutral mandate.

## Supplementary information

ESM 1 (DOCX 29 kb)

ESM 2 (DOCX 18 kb)

ESM 3 (DOCX 39 kb)

## Footnotes

1 https://www.ipcc.ch/apps/eventmanager/documents/49/230220180401-INF.10-UNFCCC.pdf

2 https://www.ipcc.ch/srccl/authors/

3 https://www.ipcc.ch/srccl/chapter/summary-for-policymakers/

4 Available at https://www.youtube.com/watch?v = KgCfmj9Vavk

5 https://www.ipcc.ch/2019/08/08/land-is-a-critical-resource_srccl/

6 Available at https://www.ipcc.ch/srccl/press/

7 Section B.6.2 included only four main conclusions mentioning the issue.

8 See https://www.youtube.com/watch?v=KgCfmj9Vavk

9 https://www.carbonbrief.org/in-depth-qa-the-ipccs-special-report-on-climate-change-and-land; https://www.eciu.net/blog/2019/ipcc-lays-out-menu-of-land-choices; https://www.sciencemediacentre.org/expert-reactionto-the-ipcc-special-report-on-climate-change-and-land/

10 Full documentation here: https://tags.hawksey.info/

11 Full documentation here: https://github.com/taspinar/twitterscraper

12 Full documentation here: https://pypi.org/project/langdetect/

13 For the purpose of protecting individual privacy, we cannot reproduce the verbatim tweet text unless the person who posted the tweet is a public figure (Williams 2012). Thus, we paraphrase and summarize the tweet text where relevant.

14 https://www.carbonbrief.org/in-depth-qa-the-ipccs-special-report-on-climate-change-and-land

15 https://www.theguardian.com/environment/2019/aug/08/climate-crisis-reducing-lands-ability-to-sustain-humanity-says-ipcc. However, an opinion article published by the paper’s columnist George Monbiot on the day of the release did strongly criticize the IPCC for not taking on the farming industry: https://www.theguardian.com/commentisfree/2019/aug/08/ipcc-land-climate-report-carbon-cost-meat-dairy

16 https://www.farminguk.com/news/farmers-criticise-media-for-biased-ipcc-reporting_53647.html

17 https://www.wsj.com/articles/vegetarianism-as-climate-virtue-signaling-11565301932

18 https://www.politico.com/news/2021/04/26/biden-not-banning-meat-usda-484609

19 See for example, the AP fact check at https://apnews.com/article/climate-business-amy-berman-jackson-beto-orourke-science-fe7c9d4a9f8f458c827677d31230f594. Several other authors also wrote articles or spoke to the media.

20 https://www.stockholmresilience.org/research/research-news/2019-11-26-a-digital-backlash-on-the-planetary-health-diet.html
