# Supplementary material for: Controversy around climate change reports: a case study of Twitter responses to the 2019 IPCC report on land
Source: Clim Change. 2021 Aug 31;167(3-4):59. doi: 10.1007/s10584-021-03182-1 (PMC8405718; doi:10.1007/s10584-021-03182-1)
Supplement: Supplementary file 1 — (DOCX 29 kb) [file 10584_2021_3182_MOESM1_ESM.docx]

**Table A Distribution of Words in Topics without ‘anchoring’**

|  | Topic 1 | Topic 2 | Topic 3 | Topic 4 | Topic 5 |
| --- | --- | --- | --- | --- | --- |
| Key words | Land, report, srccl, change, use, ipcc, models, tech, data, climate, climate hoax, fraud, average, globalist, solar | Food, need, special, meat, waste, health, protect, plant, systems, planet, help, restore, reduce, fossil, cut | Chair, working, group, fluxes, present, terrestrial, balanced, lee, low, defenders, thank, op, hoesung, tuned, debra | Emissions, carbon, sustainable, global, greenhouse, ghg, management, security, gas, warming, production, practices, degradation, soil, desertification | Indigenous, local, communities, rights, peoples, plenary, geneva, knowledge, cdnpoli, community, session, land rights, bcpoli, extreme, securing |
| Number of tweets | 5,166 | 2,296 | 1,256 | 2,480 | 1,465 |
| Toxicity | 0.112  (0.828) | 0.113 (0.0846) | 0.117  (0.0799) | 0.103  (0.0782) | 0.125  (0.0925) |
| Sentiment  Polarity | 0.135  (0.143) | 0.0942  (0.106) | 0.161  (0.178) | 0.0905  (0.114) | 0.137  (0.202) |
