# Supplementary material for: Controversy around climate change reports: a case study of Twitter responses to the 2019 IPCC report on land
Source: Clim Change. 2021 Aug 31;167(3-4):59. doi: 10.1007/s10584-021-03182-1 (PMC8405718; doi:10.1007/s10584-021-03182-1)
Supplement: Supplementary file 2 — (DOCX 18 kb) [file 10584_2021_3182_MOESM2_ESM.docx]

**Table B Selection of words for each topic**

| Topic 1 | diet | vegan | meat | consumption |
| --- | --- | --- | --- | --- |
| Topic 2 | IPCC | report | new |  |
| Topic 3 | food | land | system |  |
| Topic 4 | hoax | fake | science |  |
