# Supplementary material for: Controversy around climate change reports: a case study of Twitter responses to the 2019 IPCC report on land
Source: Clim Change. 2021 Aug 31;167(3-4):59. doi: 10.1007/s10584-021-03182-1 (PMC8405718; doi:10.1007/s10584-021-03182-1)
Supplement: Supplementary file 3 — (DOCX 39 kb) [file 10584_2021_3182_MOESM3_ESM.docx]

**Supplementary Material**

**Methods**

We used topic modelling to analyze the content of the tweets we collected. A topic model may be implemented using a variety of implementations. To date, the most common within social science is latent Dirichlet allocation (LDA). However, LDA faces many limitations, especially for shorter texts such as tweets. Thus, we opt for a different implementation introduced in Gallagher et al. (2017) called correlation explanation, or CorEx.^[[1]](#footnote-1)^

CorEx uses input data to learn the most informative set of topics in the discourse and for each input document (in this study, each tweet). CorEx calculates word concurrences across all tweets and then determines clusters of co-occurrence correlations which it then outputs as a distribution of topics. Models are scored based on the amount of information their topic distributions are able to explain about the documents in the corpus, i.e. the total correlation. Each topic is given a correlation score which reflects the amount of the model’s total correlation which can be explained by that topic. The individual topic correlation scores sum to the model’s total correlation value. Models with higher total correlation values are considered more coherent and higher performing, because each topic that the model generates is able to explain more of the information contained in the corpus. In this way, CorEx is able to identify latent topics or themes in the input discourse without needing to rely on a specific generative model.

CorEx is also able to incorporate human input in the form of ‘anchoring,’ which permits us to guide the model towards the detection of certain topics. In our analysis, we first ran the algorithm without anchors in order to determine a baseline of model performance and cohesion. We investigated the topics of this baseline model and examined in further detail the tweets assigned to each topic in order to get an initial sense of what topics emerge from the discourse and how they may relate to each other. This examination led us to develop a set of hypotheses about the emergent topics and a list of anchors to use in testing these hypotheses. As mentioned above, models with higher total correlation values more coherently capture the latent topic structure of a discourse sample. Thus, we tested various sets of anchors representing different potential explanations of our hypothesis, and recorded which anchors yielded models with the highest correlation values.

We tested several iterations with different numbers of topics, from 3 to 20, and chose the one with 5 topics as our base model, as it generated the highest total correlation value across all topics. We show the top 15 words for each topic in Table S1. This base model provided an initial indication of the prominent topics in the sample and how well the addition of ‘anchoring’ to the model might help prompt the emergence of more coherent topics.

We then calculated the number of tweets assigned to each topic to give an indication of each topic’s prevalence in the dataset, and then the average and median (in parentheses) toxicity and sentiment polarity values of the tweets assigned to each topic. Tweets can be assigned to more than one topic but with varying strengths, which explains why the sum of the row ‘numbers of tweets’ in Table S1 exceeds 6,441.

The topics of the base model lacked strong coherence, i.e. we did not observe distinct, discernible, or qualitatively sensible topics. This is also reflected by a relatively low total correlation value for the model across all topics of 4.61. Anything less than 5 indicates weak model coherence, as there are 5 topics and each receives its own correlation score which we then sum to get the total correlation score for the model.

Next, we considered several sets of words to ‘anchor’ the model. This results in a semi-supervised topic model that incorporates domain-specific knowledge to strengthen the model’s ability to uncover more nuanced aspects of the discourse (Gallagher et al., 2017). The model first tries to fit the topics using these anchors but if it is unable to, it will override the anchors to disable invalid or artificial manipulation of the topics.

Anchoring involves pre-seeding the model with key terms to help guide topic discovery. We used the base model to develop a hypothesis about the emergent topics and a list of anchors to use in testing this hypothesis. Models with higher total correlation values more coherently capture the latent topic structure of a discourse sample. Thus, we tested various sets of anchors representing different potential explanations of our hypothesis, and recorded which anchors yielded models with best performance.

We included words for each topic that we found in samples of tweets loaded to each of our tentative topics from the base model. Table S2 shows the set which yielded the model with the highest total correlation value.

We anchored only four of the five topics to allow for a ‘wildcard’ or miscellaneous topic to emerge. Building this flexibility into the model allowed us to make sure that we did not force every tweet to be allocated to one of five pre-determined topics, thereby reducing the likelihood of our anchors artificially manipulating the model based on our hypotheses. In the resulting model, the fifth topic appeared to cover miscellaneous general details pertaining to the report and its publication, as well as reminders of the press conference announcing the publication by the IPCC in Geneva.

The anchored model demonstrated a higher overall correlation of 7.91, a 72% increase over the previous baseline model. Moreover, the top words and tweets loaded to each topic also exhibited more qualitative coherence, i.e. their grouping together made sense in a way that affirmed our initial topical structure hypothesis (see Table 3 in main text). In sum, we found that the anchored correlation-based topic model produced coherent topics from the sample of English tweets.

To calculate sentiment polarity and toxicity we used the VADER sentiment analysis tool^[[2]](#footnote-2)^ and the Perspective API^[[3]](#footnote-3)^, respectively. In the VADER analysis implementation, sentiment scores range from -1 to 1, with more negative scores indicating more negative sentiment (vice versa for positive scores), and values around zero indicating neutral sentiment. Toxicity values using Perspective range from 0 to 1, with values closer to zero indicating lower toxicity and values closer to 1 indicating higher toxicity.

Two examples of tweets posted in response to the SRCCL are shown below. The first is from the London-based International Institute for Environment and Development, and is an example of negative sentiment and low toxicity:

‘It isn’t just the climate crisis that threatens poorer communities - it is also the wrong policy choices. The latest science shows the impacts of global warming - and solutions to it - risk worsening inequality if not coupled with support to the world’s poor #SRCCL.’

The calculated sentiment score of this tweet is -0.961 (i.e. it was seen as critical of the SRCCL), and toxicity score is 0.118896 meaning that the language used is not rude, disrespectful or insulting.

In contrast, a tweet posted by the UN information Centre in Beirut showed positive sentiment and low toxicity:

'The @UN #ClimateAction Summit is a moment for bold & innovative solutions, like those in the new @IPCC_CH report. Smarter policies & practices for managing our lands can help address the climate emergency & deliver substantial benefits. #SRCCL ‘

This tweet scored a high positive sentiment value of 0.935, and a low toxicity value of 0.0716.

For the purpose of protecting individual privacy, we cannot reproduce the verbatim tweet text unless the person who posted the tweet is a public figure (Williams 2012). Where possible, we included such examples but more often, we paraphrased and summarized the tweet text where relevant.

While extremely useful for processing large amounts of textual data, topic modelling faces a few important limitations. In general, it simply detects concurrences of words and phrases, and uses them to construct clusters of input documents (in our case, tweets) representing potential themes within a corpus. It does so mechanically, without knowledge of input context or of the nuances of syntax and tone. This is why we complemented our analysis with measures of toxicity and sentiment polarity, and why we manually reviewed subsets of the tweets assigned to each topic, and those at the extremes of our language measures. This additional qualitative analysis helped to make sense of the model output borne from statistical computations without higher level knowledge. Moreover, we reiterate that the dataset used in this research only represents a sample of the response to the SRCCL on Twitter due to Twitter’s data collection restrictions. From this sample, we included only English language tweets in our analysis. While most of the tweets in the sample were posted in English, we do not know if it accurately represents the full discourse including the other 40 languages used. Use of Twitter samples in studying policy and politics related issues have been criticized for its shortcoming in the past (Cihon and Yasseri, 2016).

**Table S1 Distribution of Words in Topics without ‘anchoring’**

|  | Topic 1 | Topic 2 | Topic 3 | Topic 4 | Topic 5 |
| --- | --- | --- | --- | --- | --- |
| Key words | Land, report, srccl, change, use, ipcc, models, tech, data, climate, climate hoax, fraud, average, globalist, solar | Food, need, special, meat, waste, health, protect, plant, systems, planet, help, restore, reduce, fossil, cut | Chair, working, group, fluxes, present, terrestrial, balanced, lee, low, defenders, thank, op, hoesung, tuned, debra | Emissions, carbon, sustainable, global, greenhouse, ghg, management, security, gas, warming, production, practices, degradation, soil, desertification | Indigenous, local, communities, rights, peoples, plenary, geneva, knowledge, cdnpoli, community, session, land rights, bcpoli, extreme, securing |
| Number of tweets | 5,166 | 2,296 | 1,256 | 2,480 | 1,465 |
| Toxicity | 0.112  (0.828) | 0.113 (0.0846) | 0.117  (0.0799) | 0.103  (0.0782) | 0.125  (0.0925) |
| Sentiment  Polarity | 0.135  (0.143) | 0.0942  (0.106) | 0.161  (0.178) | 0.0905  (0.114) | 0.137  (0.202) |

**Table S2 Selection of words for each topic**

| Topic 1 | diet | vegan | meat | consumption |
| --- | --- | --- | --- | --- |
| Topic 2 | IPCC | report | new |  |
| Topic 3 | food | land | system |  |
| Topic 4 | hoax | fake | science |  |

1. https://github.com/gregversteeg/corex_topic [↑](#footnote-ref-1)
2. Full documentation here: <https://github.com/cjhutto/vaderSentiment> [↑](#footnote-ref-2)
3. Full documentation here: <https://github.com/conversationai/perspectiveapi/blob/master/api_reference.md> [↑](#footnote-ref-3)
